# A Retrospective Study of the Profiles of Outpatients Attending the State Adolescent Friendly Health Resource Center, Andhra Pradesh, South India

**DOI:** 10.7759/cureus.69878

**Published:** 2024-09-21

**Authors:** Navya Krishna Naidu, Rajeev Aravindakshan, Venkatashiva Reddy B, Vijayan Sharmila, Arti Gupta

**Affiliations:** 1 Department of Community and Family Medicine, All India Institute of Medical Sciences, Mangalagiri, Guntur, IND; 2 Department of Obstetrics and Gynaecology, All India Institute of Medical Sciences, Mangalagiri, Guntur, IND

**Keywords:** adolescents, healthcare, morbidity, nutrition, telemedicine

## Abstract

Background: Adolescents compose a significantly large age group, and attention to their health needs is lacking due to insufficient data and policy implementation globally and in India. India, having 268 million adolescents, faces the world's largest adolescent population, which highlights the importance of addressing their health requirements. The aim of this study was to describe the morbidity profiles of adolescent patients attending the State Adolescent Friendly Health Resource Centre at a tertiary-level healthcare institution in South India.

Methodology: A retrospective secondary data analysis was conducted at the State Adolescent Friendly Health Resource Center from 2022 to 2023. Data on adolescent health were collected from this center's OPD using a structured questionnaire, which included information on demographics, immunization status, chief complaints, and dietary intake. Diagnosis coding was done using the International Classification of Diseases, Tenth Revision (ICD-10), with estimated energy and protein intake.

Results: The study analyzed 4,000 adolescent patients, with a nearly equal gender distribution across physical OPD and telemedicine services. Adolescent females between 15 and 16 years had more visits for consultation. Most patients were within the normal BMI range, and telemedicine was popular among females with normal BMI. Obesity was more common in males, while overweight was prevalent in females. Energy and protein intake varied across BMI categories, with obese individuals having the highest intake. The morbidity profiles revealed significant gender differences in healthcare utilization and disease patterns.

Conclusion: Gender differences in adolescent morbidity and the underutilization of telemedicine highlight the need for tailored healthcare interventions and further research based on socioeconomic factors.

## Introduction

Adolescence encompasses the period of development between childhood and adulthood, typically ranging from ages 10 to 19 years [1]. Adolescence marks a pivotal life stage characterized by the growth and maturation of all bodily organs and physiological systems. This phase is accompanied by shifts in health needs owing to unique biological, psychological, and social factors [2]. According to the 2011 census report, adolescents make up 21% of India's population [3]. India, with 268 million adolescents, boasts the world's largest adolescent population, where every fifth individual falls within the age range of 10 to 19 years [2,4,5]. The state of Andhra Pradesh has approximately nine million adolescents within its population of 49 million, equating to one-fifth of its total inhabitants falling within the 10-19 age range [6]. 

Adolescents constitute a significant and expanding segment of the global population, particularly in developing nations. Their distinct health requirements often go unnoticed due to insufficient data from healthcare institutions and the lack of implementation of adolescent health policies [7]. Globally and within India, there has been an increasing commitment to child and adolescent health over the past decade. The substantial and rising proportion of adolescents in India underscores the urgency of addressing their health and developmental requirements to enhance overall population health and well-being [8-11]. Understanding the morbidity burden among adolescents is crucial for recognizing their healthcare needs [2,10,11]. Unfortunately, such data are scarce in developing countries, prompting our study to outline the morbidity patterns among adolescent patients in the State Adolescent Friendly Health Resource Centre of our healthcare facility. The objective of the study was to profile the morbidity pattern of adolescents consulted at the outpatient department (OPD) physically or virtually in the State Adolescent Friendly Health Resource Centre.

## Materials and methods

This is a retrospective secondary data analysis study carried out in the OPD of the Rashtriya Kishor Swasthya Karyakram (RKSK)-supported State Adolescent Friendly Health Resource Center, UNICEF PARC (Establishment of the Pediatric Adolescent Resource Center funded by the United Nations Children's Fund, Hyderabad office, India) project of the Department of Community and Family Medicine (CFM), All India Institute of Medical Sciences (AIIMS), Mangalagiri, Andhra Pradesh, India in 2022-2023. An adolescent-friendly health OPD is managed by two project staff and supported by faculty in the OPD, Department of CFM, AIIMS Mangalagiri, providing services six days a week. The data were collected from adolescents who were physically attending and using the telemedicine facility at the center's OPD. The telemedicine facility at AIIMS Mangalagiri comprises the in-built HMIS (Health Management Information System) of AIIMS Mangalagiri, the eSanjeevani OPD (National Telemedicine Service of India), and telecalling services. In addition to history taking and case-specific counseling for adolescents, this center provides the following services for adolescent care: first, nutrition counseling based on a 24-hour dietary recall, with data entered into the DietCal software (ProfoundTech Solutions, New Delhi, India) to estimate energy and protein intake routinely for every adolescent; second, assessment of immunization status for the Td vaccine; and third, anthropometric measurements such as height, weight, and Body Mass Index (BMI).

Adolescent data were retrieved using a questionnaire with patient demographics, such as age, gender, immunization of tetanus-diphtheria (Td) vaccine at 10 or 16 years as per their age, chief complaints, weight, height, ICD-10 code of diagnosis, energy intake in 24 hours and protein intake in 24 hours. BMI-for-age was assessed using the WHO growth reference data for children and adolescents aged five to 19 years, which provides age and gender-specific BMI Z-scores to evaluate nutritional status [12]. The classification is as follows: severe thinness (BMI-for-age Z-score < -3 standard deviation (SD)), thinness (BMI-for-age Z-score between -3 SD and -2 SD), normal (BMI-for-age Z-score between -2 SD and +1 SD), overweight (BMI-for-age Z-score between +1 SD and +2 SD), and obesity (BMI-for-age Z-score > +2 SD).

The data regarding BMI were recorded as the BMI-for-age 5-19 years (Z-scores) with labels charts for both genders. The data for anthropometry using the telemedicine facility were collected by enquiring the informant (parent/caregiver) about the approximate values of height and weight of the adolescent as per the recent school records noted by the informant. Two doctors independently coded the diagnosis according to the International Statistical Classification of Diseases and Related Health Problems, Tenth Revision (ICD-10) Version for 2010 [13]. For estimating energy and protein intake in 24 hours of diet, the DietCal software was used and the estimated values from the software were compared with the recommended guidelines by the Indian Council of Medical Research - National Institute of Nutrition (ICMR - NIN). Data entry was done using Microsoft Excel 2010 (Microsoft Corporation, Redmond, Washington, United States), and analysis was done using IBM SPSS Statistics for Windows, Version 28, (Released 2021; IBM Corp., Armonk, New York, United States). Descriptive analysis was done and the chi-square test was used to compare proportions as applicable. For all analyses, statistical significance was determined by a p-value of < 0.05. Informed consent waiver and approval for the study were obtained from the All India Institute of Medical Sciences, Mangalagiri, Ethics Committee (AIIMS/MG/IEC/2023-24/41 dated 12-10-2023).

## Results

**Figure 1:**
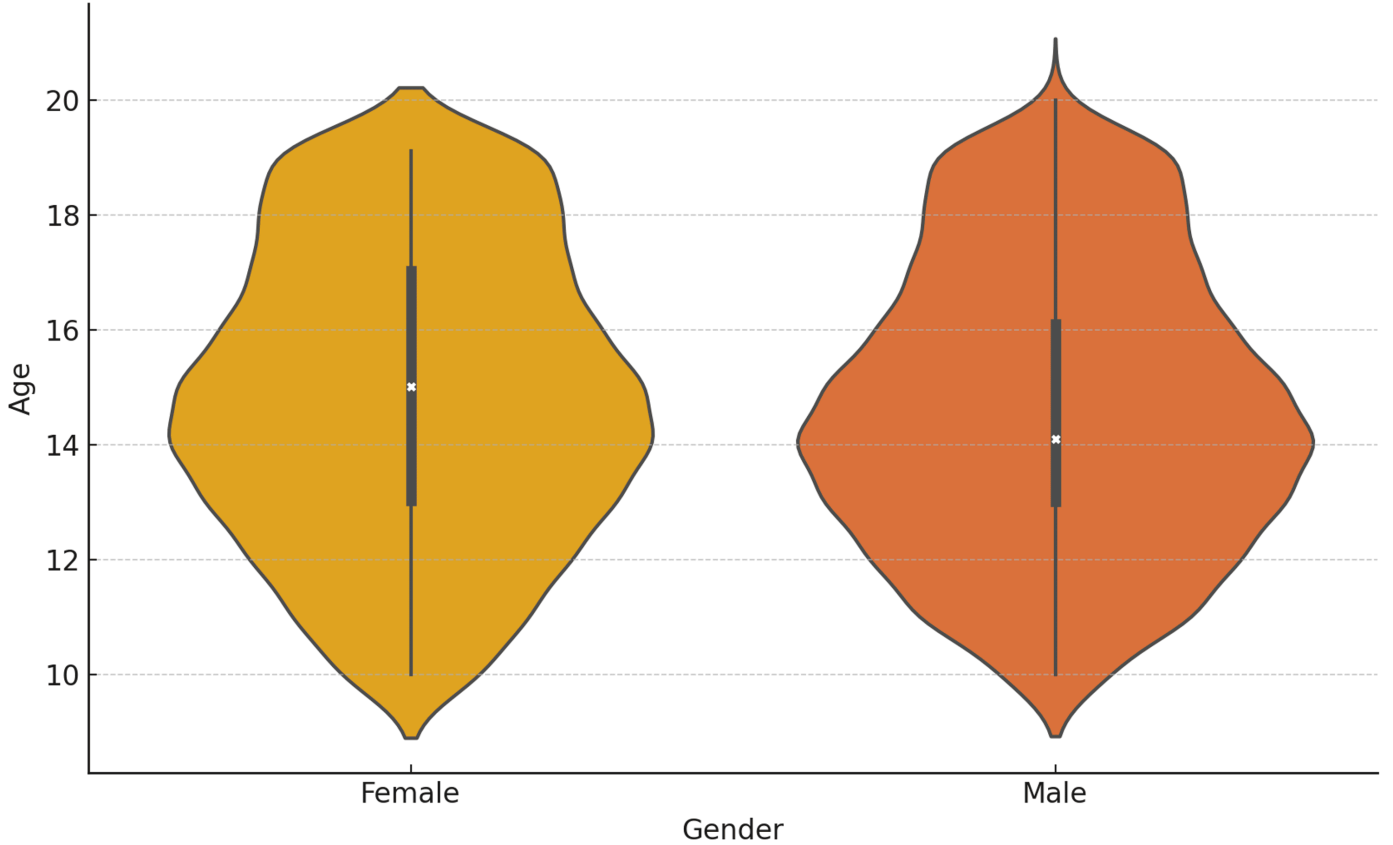
Gender-wise distribution of age of the study population

A total of 4000 adolescent patients aged 10-19 years were analyzed, with the distribution between adolescent males and females nearly balanced in both physical OPD and telemedicine services. There was a noticeable increase in females around ages 15-16 years (Figure 1).

The median (IQR) weight and height among adolescent males were 48 (19.48) kgs and 159 (18) cms, respectively, while among adolescent females were 44.7 (15.27) kgs and 151.5 (10) cms, respectively. The gender distribution in Table 1 shows a slight predominance of females in physical OPD (1028, 25.70%) and male predominance in telemedicine/telecalling consultations (1041, 26.03%). When examining nutrition status based on BMI, the data revealed significant differences between physical OPD (χ² value: 29.54, p-value <0.001) and telemedicine/telecalling consultations (χ² value: 16.20, p-value <0.05). Obesity rates were higher in physical OPD (80, 29.85% males and 78, 29.10% females) compared to telemedicine (66, 24.63% males and 44, 16.42% females). The overweight category showed 135 (22.02%) males and 187 (30.51%) females in physical OPD, while telemedicine had 147 (23.98%) males and 144 (23.49%) females.

**Table 1:** Demographic and health characteristics of the study population (n=4000) ^*^There are 261 cases with missing data for COVID-19 status. ^#^There are 340 cases with missing data for immunization status for the tetanus-diphtheria (Td) vaccine

		Physical OPD				Telemedicine/telecalling				
		Male	Female	χ² value	p-value	Male	Female	χ² value	p-value	Total
		N (%)	N (%)			N (%)	N (%)			N (%)
Gender		959 (23.98)	1028 (25.70)			1041 (26.03)	972 (24.30)			4000 (100)
Nutrition status (BMI)	Obesity	80 (29.85)	78 (29.10)	29.54	<0.001	66 (24.63)	44 (16.42)	16.20	<0.05	268 (6.70)
	Overweight	135 (22.02)	187 (30.51)			147 (23.98)	144 (23.49)			613 (15.35)
	Normal	569 (22.55)	652 (25.84)			641 (25.41)	661 (26.20)			2523 (63.07)
	Thinness	126 (28.06)	91 (20.27)			137 (30.51)	95 (21.16)			449 (11.22)
	Severe thinness	49 (33.33)	20 (13.61)			50 (34.01)	28 (19.05)			147 (3.67)
COVID-19^*^	Yes	26 (32.91)	32 (40.51)	0.48	0.48	13 (16.46)	8 (10.13)	0.85	0.35	79 (2.11)
	No	852 (23.28)	870 (23.77)			1004 (27.43)	934 (25.52)			3660 (98.89)
Immunization status for the Td vaccine at 10 and 16 years^#^	Yes	456 (19.04)	430 (17.95)	3.15	0.20	798 (33.32)	711 (29.69)	12.35	<0.05	2395 (65.44)
	No	86 (24.86)	102 (29.48)			91 (26.30)	67 (19.36)			346 (9.45)
	Don't know	310 (33.73)	338 (36.78)			115 (12.51)	156 (16.97)			919 (25.11)

The majority of individuals fell under the normal BMI category (2523, 63.07%), with similar distributions across both types of consultation. Thinness and severe thinness were more prevalent in the telemedicine services for both genders. For COVID-19 infection, the rates were low in both groups, with a slightly higher prevalence in females using physical OPD (32, 40.51%) compared to males (26, 32.91%). The χ² value was 0.48 with a p-value of 0.48 for physical OPD and 0.85 with a p-value of 0.35 for telemedicine, indicating no significant difference. Immunization status for the Td vaccine showed varied results. A higher percentage of individuals in telemedicine (798, 33.32% males and 711, 29.69% females) were immunized showing a significant difference (χ² value: 12.35, p-value <0.05) compared to those in physical OPD (456, 19.04% males and 430, 17.95% females) showing no difference (χ² value: 3.15, p-value = 0.20). The 'don't know' category for the Td vaccine was notably higher in physical OPD (310, 33.73% males and 338, 36.78% females) compared to telemedicine (115, 12.51% males and 156, 16.97% females).

**Figure 2:**
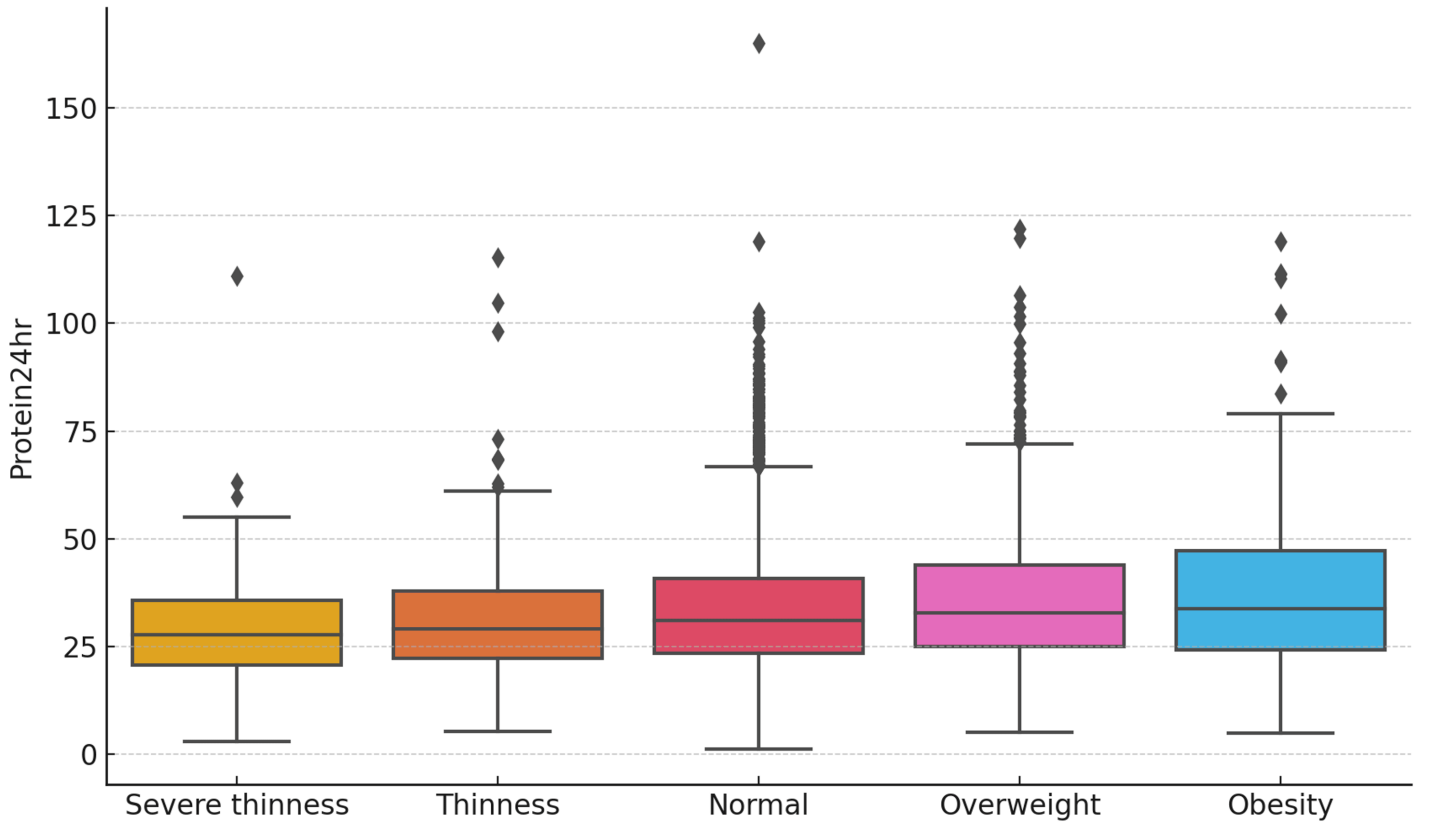
Distribution of 24-hour protein intake (g/day) across different nutritional status categories

Furthermore, the dietary intake assessment using the DietCal software was conducted for 3962 individuals. The median (IQR) energy intake over a 24-hour period among adolescents was 1134.25 (453.18) kcal. Similarly, the median (IQR) protein intake was 31.10 (17.40) grams. Obese individuals had the highest mean ± SD) energy intake (1258.96 ± 526.5 kcal/day), followed by overweight individuals (1235.98 ± 416 kcal/day). Those with severe thinness had the lowest mean ± SD energy intake (1049.65 ± 432.6 kcal/day), while normal-weight individuals' average was 1162.31 ± 379.5 kcal/day. Likewise, the mean ± SD protein intake was highest among obese individuals at 38.04 ± 20.05 g/day, followed by overweight individuals who consumed an average of 36.58 ± 17.27 g/day. Individuals with severe thinness had the lowest protein intake at 29.47 ± 14.05 g/day, while those with normal weight had a mean intake of 34.78 ± 15.47 g/day, as illustrated in Figure 2.

**Figure 3:**
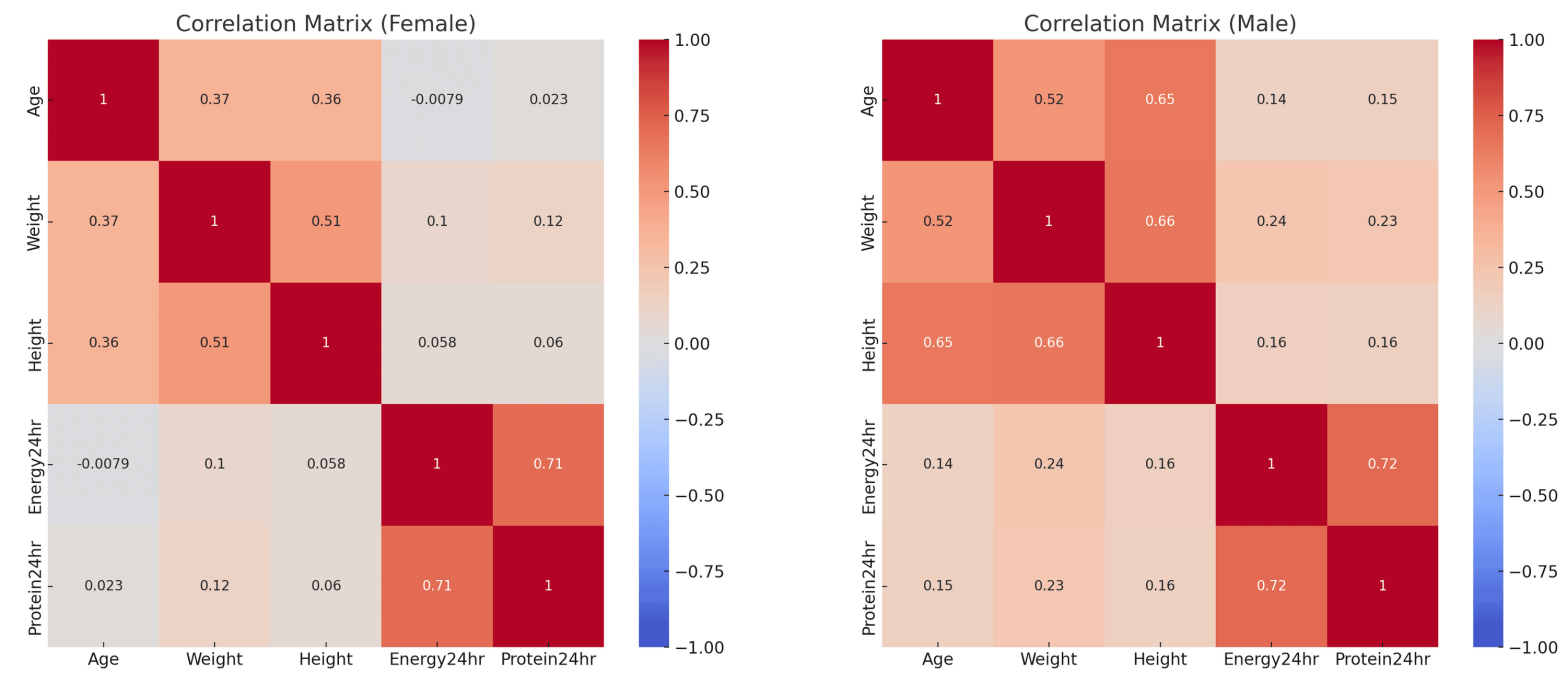
Gender-wise correlation heatmap of age, weight, height, energy, and protein intake

The heatmap in Figure 3 displays correlations between five variables: age, weight, height, energy intake in 24 hours (kcal/day), and protein intake in 24 hours (grams/day), with separate matrices for females (left) and males (right). The correlations between age and both height and weight were notably stronger in males (0.65 and 0.52, respectively) compared to females (0.36 and 0.37), indicating gender differences in the relationship between age, physical characteristics, and nutritional intake. While the correlation between weight and height was also higher in males (0.66) than in females (0.51); males appeared to exhibit continued growth and increased nutritional needs with age, whereas females showed more stability. The mean energy intake (Energy24hour) and mean protein intake (Protein24hour) correlation was similar for both genders but slightly stronger in males (0.72 vs. 0.71), suggesting a consistent relationship between energy and protein intake regardless of gender.

Table 2 shows the morbidity profiles of the adolescent patients comparing the distribution of ICD-10 codes between physical and telemedicine consultations, focusing on gender differences. For most categories, no significant gender differences were found between the two consultation types. However, notable differences were observed in certain areas such as endocrine, nutritional, and metabolic diseases (E00-E89); physical consultations had 144 males (40.3%) and 213 females (59.7%), whereas telemedicine had 32 males (65.3%) and 17 females (34.7%), showing a significant difference (χ² value: 10.94, p < 0.05). Diseases of the respiratory system (J00-J99), with physical consultations having 115 males (57.2%) and 86 females (42.8%), and telemedicine having 103 males (71.5%) and 41 females (28.5%) also showed a significant difference (χ² value: 7.39, p < 0.05). For diseases of the digestive system (K00-K95), physical consultations had 251 males (55.3%) and 203 females (44.7%), compared to telemedicine with 17 males (31.5%) and 37 females (68.5%), showing a significant difference (χ² value: 10.97, p < 0.05).

**Table 2:** Morbidity profiles of outpatients of a State Adolescent Friendly Health Resource Centre in South India (n=4000) ICD-10: International Statistical Classification of Diseases and Related Health Problems, Tenth Revision, Version for 2010

ICD-10 codes	Physical consultation			Telemedicine consultation			χ² value	p-value
	Male	Female	Total	Male	Female	Total		
	N (%)	N (%)	N (%)	N (%)	N (%)	N (%)		
Certain infectious and parasitic diseases (A00-B99)	42 (51.9)	39 (48.1)	81 (4.1)	7 (53.8)	6 (46.2)	13 (0.6)	0.02	0.89
Diseases of the blood and blood-forming organs and certain disorders involving the immune mechanism (D50-D89)	5 (45.5)	6 (54.5)	11 (0.6)	2 (20)	8 (80)	10 (0.5)	1.53	0.36
Endocrine, nutritional, and metabolic diseases (E00-E89)	144 (40.3)	213 (59.7)	357 (18.0)	32 (65.3)	17 (34.7)	49 (2.4)	10.94	<0.05
Diseases of the nervous system (G00-G99)	81 (31.5)	176 (68.5)	257 (12.9)	24 (33.3)	48 (66.7)	72 (3.6)	0.08	0.77
Diseases of the eye and adnexa (H00-H59)	11 (57.9)	8 (42.1)	19 (1.0)	9 (60)	6 (40)	15 (0.7)	0.02	0.90
Diseases of the ear and mastoid process (H60-H95)	30 (66.7)	15 (33.3)	45 (2.3)	2 (33.3)	4 (66.7)	6 (0.3)	2.52	0.11
Diseases of the circulatory system (I00-I99)	26 (46.4)	30 (53.6)	56 (2.8)	1 (20)	4 (80)	5 (0.2)	1.30	0.37
Diseases of the respiratory system (J00-J99)	115 (57.2)	86 (42.8)	201 (10.1)	103 (71.5)	41 (28.5)	144 (7.2)	7.39	<0.05
Diseases of the digestive system (K00-K95)	251 (55.3)	203 (44.7)	454 (22.8)	17 (31.5)	37 (68.5)	54 (2.7)	10.97	<0.05
Diseases of the skin and subcutaneous tissue (L00-L99)	82 (58.2)	59 (41.8)	141 (7.1)	16 (57.1)	12 (42.9)	28 (1.4)	0.10	0.92
Diseases of the musculoskeletal system and connective tissue (M00-M99)	50 (53.2)	44 (46.8)	94 (4.7)	9 (60)	6 (40)	15 (0.7)	0.24	0.62
Diseases of the genitourinary system (N00-N99)	4 (33.3)	8 (66.7)	12 (0.6)	2 (66.7)	1 (33.3)	3 (0.1)	1.11	0.53
Symptoms, signs, and abnormal clinical and laboratory findings, not elsewhere classified (R00-R99)	7 (63.6)	4 (36.4)	11 (0.6)	2 (100)	0 (0)	2 (0.1)	1.05	1.00
Health checkup	92 (46.9)	104 (53.1)	196 (9.9)	802 (51.1)	768 (48.9)	1570 (78.0)	1.20	0.27
Others	19 (36.5)	33 (63.5)	52 (2.6)	13 (48.1)	14 (51.9)	27 (1.3)	0.99	0.32
Total	959 (48.3)	1028 (51.7)	1987 (100)	1041 (51.7)	972 (48.3)	2013 (100)		

## Discussion

The State Adolescent Friendly Health Resource Centre, established in 2022 under the Department of Community and Family Medicine at AIIMS, Mangalagiri, supported by the RKSK and the National Health Mission (NHM), and funded by UNICEF and CDC-PEPFAR, plays a pivotal role in capacity building, patient care, community engagement, needs assessment, and research, focusing on delivering adolescent-friendly health services.

The present analysis was focused on adolescents who visited the State Adolescent Friendly Health Resource Center in 2022-23. In this study, overweight and obesity were observed in 15.35% and 6.70% of adolescents, respectively. Notably, severe thinness was found in 3.67% of cases, with a higher incidence among males. This contrasts with another Indian study, which reported prevalences of 4.8% for overweight, 1.1% for obesity, and 24.4% for thinness in this population [14]. COVID-19 positivity was 2.11% among adolescents during our study period, whereas two large multicentric studies in India identified higher rates of 55.7% and 61.6% among young people and adolescents [15,16]. The vaccination coverage for the Td vaccine among 10- and 16-year-olds in our sample was 65.44%; intriguingly, comparable statistics for the adolescent population are scarce both within India and globally. Furthermore, median daily energy and protein intakes were recorded at 1134.25 (453.18) kcal and 31.10 (17.40) grams, respectively, which were lower than those found in an international study among adolescents, where mean (±SD) daily intakes were 1517 (±644) kcal for calories and 94.77 (±71.87) grams for protein [17].

In this study, data were collected from both physical and telemedicine consultations among adolescents. Despite its widespread use during the COVID-19 pandemic, telemedicine is now being applied for medical and psychiatric care, as well as follow-up appointments, for adolescents [18]. Our findings indicate a notable non-utilization of telemedicine services for these conditions within the study population. This contrasts with similar studies where adolescents showed a preference for in-person healthcare visits, citing engagement and confidentiality concerns with telemedicine [19].

The most common diagnoses in our study population included diseases of the digestive system (physical: 22.8%, telemedicine: 2.7%), followed by endocrine, nutritional, and metabolic diseases (physical: 18.0%, telemedicine: 2.4%), diseases of the respiratory system (physical: 10.1%, telemedicine: 7.2%), diseases of the nervous system (physical: 12.9%, telemedicine: 3.6%), respectively. This differs from the findings in a Nigerian study, where nervous system disorders (18.4%), respiratory disorders (11%), and diseases of blood products (9.6%) were prevalent [7]. Comparable research from Portugal identified endocrine, nutritional, and metabolic diseases (34%), followed by mental and behavioral disorders (32%), as the most frequent diagnoses [20]. An Indian study highlighted that the most common diagnosis was related to menstruation (46.29%), sexual and reproductive health (28.19%), nutrition (5.91%), and mental health (1.67%) [21].

This study offers a concise overview of the morbidity profiles of adolescents availing services both physically and via telemedicine over a one-year period. One prominent advantage of this study lies in its robust sample size, providing a reliable estimate of the broader spectrum of adolescent health issues. This information will aid in designing tailored services to address patient needs and in training healthcare staff to better meet these requirements. This would help policymakers and researchers to strengthen the adolescent health system. Understanding the morbidity profiles is crucial for delivering timely and effective treatment to the community, as well as for improving the quality of public health services.

A limitation identified was the inability to comprehensively measure all conceivable health dimensions pertinent to adolescents and young adults. The present analysis was centered on data derived from an adolescent health center in South India, thus limiting its generalizability to the entirety of India. While the morbidity profiles were stratified by gender, the classification based on socioeconomic status was not feasible. A limitation of this study was the use of the 24-hour dietary recall method, which may not have provided an entirely accurate representation of dietary intake due to potential recall bias and day-to-day variability in food consumption. Anthropometric data recorded on telemedicine consultations was a limitation as it may have led to minor variations in study results. The missing data were not analyzed, which could have introduced minor variations in the findings. The study was managed by two project staff, occasionally resulting in misclassification during coding. However, the study's robustness is bolstered by the inclusion of a substantial number of illness episodes, enhancing the credibility of its findings.

## Conclusions

This study offers crucial insights into the morbidity patterns and healthcare utilization among adolescents, showing significant gender differences and underutilization of telemedicine for specific conditions. The findings highlight the need for tailored healthcare interventions and policy adjustments to address the diverse health challenges faced by this population. In spite of its limitations, the abundant sample size increases the reliability of the results and emphasizes the importance of targeted strategies to enhance adolescent health services. Future studies should investigate socioeconomic factors and a wider geographic scope in order to further enhance health interventions for teenagers.
